# Co-designing a web-based intervention (RESTORE) to support self-management of cancer-related fatigue in people living with a brain tumour

**DOI:** 10.1007/s00520-025-09471-0

**Published:** 2025-04-28

**Authors:** R. Campbell, J. M. Shaw, H. Banks, T. Carlick, M. M. Faris, M. S. Jeon, D. Legge, C. Foster, R. Leonard, R. J. Chan, M. R. Agar, A. Miller, H. M. Dhillon, the BRAINS Investigator Group, Thea Blackler, Georgia Halkett, Helen Haydon, Brian Kelly, Eng-Siew Koh, Anna Nowak, Tamara Ownsworth, Kerryn Pike, Nicole Rankin, Ursula Sansom-Daly, Joel Rhee, Kristi Milley, Katarzyna Lion, Jill Chen, Tiffany Fazon, Melinda Lyne, Sian Virtue-Griffiths, Kathryn Vitangcol, Jessica Buster, Emma McDougall

**Affiliations:** 1 Psycho-Oncology Cooperative Research Group, School of Psychology, Faculty of Science, The University of Sydney, Sydney, NSW Australia; 2 Oliva Newton-John Cancer and Wellness Centre, Austin Hospital, Heidelberg, Australia; 3 Curtin School of Nursing/Curtin Health Innovation Research Institute, Faculty of Health Sciences, Curtin University, Bentley, Australia; 4 Centre for Psychosocial Research in Cancer (CentRIC), School of Health Sciences, University of Southampton, Southampton, UK; 5 Brain Cancer Collective, NHMRC Clinical Trials Centre, University of Sydney, Sydney, NSW Australia; 6 Caring Futures Institute, College of Nursing and Health Sciences, Flinders University, Bedford Park, SA Australia; 7 Improving Palliative, Aged and Chronic Care Through Clinical Research and Translation (IMPACCT), Faculty of Health, University of Technology Sydney, Broadway, NSW Australia

**Keywords:** Cancer-related fatigue, Primary brain tumour, Qualitative research, Co-design, Online intervention

## Abstract

**Purpose:**

Cancer-related fatigue (CRF) is a debilitating symptom commonly reported by people with a brain tumour (BT). Many interventions have been developed to reduce CRF; however, few have been evaluated in people with BT despite the unique functional deficits experienced by this population. We aimed to explore the appropriateness of a web-based intervention (RESTORE) to support self-management of fatigue for people with a BT and identify recommended modifications.

**Methods:**

Semi-structured interviews were conducted with people with BT, their caregivers and healthcare professionals (HCPs) who treat them. Interviews explored the appropriateness of RESTORE for this population, and suggested modifications to improve relevance and suitability. Interviews were transcribed and analysed thematically using interpretive description to devise recommendations.

**Results:**

Forty participants were interviewed (24 people with BT, 5 caregivers, 11 HCPs). Four themes were identified: feedback on content; feedback on format; feedback on use; and, barriers to engagement. These themes were linked by an overarching need for flexible and responsive tailoring to the unique needs of people with BT. Thirty-two recommended modifications were derived from feedback to optimise RESTORE for this population.

**Conclusion:**

Results suggest a BT-specific version of RESTORE would be acceptable to address fatigue in this population. Recommended adaptations include greater flexibility and tailoring of content and format for effective use among people with BT. Barriers to engagement including digital access and literacy and awareness of the resource should be addressed in the implementation of a BT-specific version of RESTORE.

**Supplementary Information:**

The online version contains supplementary material available at 10.1007/s00520-025-09471-0.

## Introduction

Primary brain tumours are relatively rare cancers causing high morbidity and mortality with an overall 5-year survival rate of 22% [1]. People surviving a brain tumour (BT) diagnosis can experience changes in brain structure and function due to the disease and its treatments, causing a diverse range of symptoms, behaviours and deficits. These contribute to cancer-related fatigue (CRF), one of the most common and distressing physical symptoms reported by BT survivors [2–4]. CRF is defined as ‘a distressing, persistent, subjective sense of physical, emotional and/or cognitive tiredness or exhaustion related to cancer or cancer treatment, that is not proportional to recent activity and interferes with usual functioning’ [5]. Approximately 50% of people with high-grade gliomas report CRF after initial surgery [6], and up to 94% report CRF following tumour recurrence [4]. Of those with low-grade gliomas, between 39 and 77% report CRF [7], with 39% reporting severe CRF up to 8 years after completing treatment [8]. These high prevalence rates are concerning as CRF can have a profound negative impact on people’s ability to engage in their social, daily and self-management activities and impair overall quality of life [3, 9].

Despite these negative impacts, CRF is often underdiagnosed and undertreated in people with cancer [10–12]. Many lifestyle, psychosocial and pharmacological interventions targeting CRF have been evaluated in breast or other cancer populations, with International guidelines recommending exercise, cognitive behavioural therapy (CBT) and mindfulness-based programs to reduce the severity of fatigue both during and after cancer treatment [13]. Yet very few interventions have been evaluated in people with BT [14]. A Cochrane review found those that have focused exclusively on pharmacological approaches, finding no evidence for their effectiveness [14]. Thus, there is a need to evaluate non-pharmacological interventions with established efficacy in this population, to help BT survivors manage their CRF, improve their quality of life and reduce disease morbidity. However, although CRF experienced by people with BT and other cancer types is multifactorial [15, 16], the unique sensory-motor, memory and attention processing deficits experienced by people with BT likely necessitates adaptation of existing non-pharmacological interventions, prior to effectiveness testing, to tailor interventions to meet their unique needs [17, 18].

Online interventions have many potential benefits in terms of cost-effectiveness, convenience and accessibility to patients [19]. RESTORE is a web-based intervention designed to enhance self-efficacy to self-manage CRF following primary cancer treatment created by the Macmillan Survivorship Research Group (now Centre for Psychosocial Research In Cancer: CentRIC^+^) in the UK [20]. RESTORE comprises five sessions informed by cognitive behavioural therapy, self-efficacy theory, existing CRF patient resources and evidence of CRF management in cancer survivors [20]. Within RESTORE, a cancer survivor is defined as anyone who has completed primary cancer treatment. Sessions are recommended to be completed weekly in consecutive order, each taking approximately 30 min. See Supplementary File 1 for an overview of the intervention content and sessions.

An evaluation of RESTORE confirmed feasibility and demonstrated preliminary efficacy in enhancing self-efficacy to self-manage CRF in a mixed sample of cancer survivors [21]. However, this evaluation did not include any people with BT. A recent review of online self-management resources for adults living with malignant BT identified the need for resources that better address patient needs related to rehabilitation that do not require a high level of cognitive ability [22]. The potential benefits of online resources in terms of accessibility and reach warrant further investigation of their appropriateness for people with BT, and how they can be optimised to better support this population. Given the unique deficits experienced by people with BT [17, 18], exploration of the appropriateness of RESTORE for this population is warranted, as well as identification of any necessary modifications to enhance its usefulness. RESTORE was selected for examination in this population because its development was informed by CBT (an approach recommended by International CRF guidelines [13]); it is designed to target CRF as a primary outcome; it is readily available in English (required for the Australian context); and there is evidence to support its feasibility and preliminary efficacy [21].

Co-design is a collaborative approach to intervention development in which the end-user provides their lived experience expertise to inform the design process to ensure the intervention meets all stakeholder needs [23]. Co-designed interventions are more likely to be acceptable to both providers and end-users, facilitating adoption and sustainability in practice [23]. We aimed to conduct the preliminary steps of a co-design approach with people with BT, their caregivers and healthcare professionals providing care to people with BT by (i) exploring perceptions of the appropriateness of the RESTORE intervention and (ii) eliciting feedback about recommended modifications to tailor suitability of RESTORE for this population.

## Methods

### Study design

This qualitative study used thematic analysis with interpretive description methodology [24] to explore perceptions of the RESTORE intervention for people with BT. This inductive approach acknowledges the variety of experiences within health systems rendering data saturation not relevant [25]. This study was approved by The University of Sydney Human Research Ethics Committee (Project number: 2022/374), and conducted in accordance with the NHMRC National Statement on Ethical Conduct in Human Research 2023.

The interpretive description methodology is an inductive approach often used in health research [24]. Through use of informed questioning and reflexivity, the subjective experiences and opinions of participants can be critically examined to inform changes to disciplinary thought and practice [26, 27]. In the context of the current study, we used this approach to explore and examine the opinions of people with BT, caregivers and healthcare professionals (informed by their personal/professional experiences) to develop modifications to an existing intervention (RESTORE).

### Participants

We recruited three groups of participants: (1) people with BT; (2) primary caregivers of people with BT; and, (3) healthcare professionals (HCPs) experienced in caring for them. Participants were recruited through social media, and email invitations to investigators’ professional networks and relevant organisations, and promotion in public fora. BT representatives were included if they had lived experience with primary BT (i.e., as a patient or primary caregiver) and lived in Australia. HCPs were eligible if they had experience providing care to adults (≥ 18) with BT in Australia or New Zealand. All interviews were conducted in English. This sample was also used for a parallel study conducted by the author group [3].

### Measures

Demographic and clinical/caregiving/professional characteristics of participants were collected using an online Qualtrics survey [28]. Feedback on RESTORE was explored using semi-structured interviews. Participants were asked open-ended questions about the content and format of RESTORE, and their views on how it would need to be tailored and implemented for people with BT (Supplementary File 2–Part 2).^1^

### Procedure

The online study invitation was distributed through relevant recruitment channels and networks (e.g. National Cooperative Trials Group for Neuro-Oncology trials [COGNO], Peace of Mind Foundation, and Brain Tumour Alliance Australia) and included a link to the participant information sheet explaining the study purpose, consent form, and demographics survey. After consent was obtained, participants were emailed a 10-min video providing an overview of the content of RESTORE and a link to access the intervention. All participants were required to watch the video and review at least one of five RESTORE sessions; if participants had not done this, it was shown to them during the interview prior to engaging in the interview questions.

Semi-structured interviews were conducted via online videoconference by members of the research team (RC, PhD, female; HB, BPsych Honours, female; or TC, BPsych Honours, Male). All interviewers were employed in full-time research positions at the University of Sydney, trained in psychological and qualitative research methods and had no prior relationship with interviewees. Only the interviewer and interviewee were present during the interviews and interviews were audio-recorded. Member checking interviews were conducted with people with BT, who had not previously participated in interviews, to assess whether the generated themes and proposed modifications accurately reflected their opinions and experiences. Participants were not able to add, edit, or delete data; rather, they provided comment on whether results matched their experience.

### Data analysis

Quantitative survey data were reported using descriptive statistics. Categorical data were reported as percentages and continuous data as medians and interquartile ranges (IQR).

Qualitative data were analysed using thematic analysis with an interpretive description lens. This involved a process of data familiarisation, coding, generating and refining themes. The research team then used interpretive description to critically examine the meaning and implications of the themes to derive recommended modifications to the RESTORE intervention. This involved the research team using their own knowledge and experience of the research context to collaboratively interpret the themes and subthemes via team discussions to devise recommended modifications.

Trint [29] audio transcription software was used to generate verbatim transcripts of interview audio-recordings. Once checked for accuracy against audio-recordings and de-identified, three transcripts were selected at random for preliminary analysis. The research team (RC, HB, TC, MF, MJ, HD and JS) independently reviewed transcripts and applied preliminary codes to passages of potential relevance. Based on these transcripts, researchers collaboratively developed a coding framework of clearly described themes and subthemes. The coding framework was iteratively refined and applied to all transcripts using NVIVO [30]. Any discrepancies in coding or interpretations were discussed as a team until consensus was reached. Reflexivity was ensured through iterative analysis, collaborative reflection as a team, and member checking interviews.

Reporting adhered to the 32-item consolidated criteria for reporting qualitative research [31] (COREQ; Supplementary File 3).

## Results

### Demographic and clinical characteristics

A total of 40 participants completed the survey and participated in an interview. The median interview length was 41 min (IQR = 14). The median age was 51 years (IQR = 14.8).

Among people with BT (*n* = 24), there was a relatively even spread in gender, grade of diagnosis, and time since diagnosis. The most common diagnosis was glioma (58%). Most received surgery (63%), chemotherapy (54%), or radiation therapy (54%), with many (46%) receiving a combination of treatments. All participating caregivers (*n* = 5) were female and cared for their spouse, with a median age of 52 (IQR = 9.5). Most (60%) cared for someone with a glioma, with the length of care provided ranging from ≤ 2 years to ≤ 8.

Among HCPs (*n* = 11), a range of clinical specialties and experience working with people with BT were represented. Most were female (82%) and worked in an outpatient hospital setting (73%). The typical patient profile seen by HCPs was someone with glioblastoma multiforme (91%) between the ages of 50–65 (73%).

Tables 1 provides an overview of patient and HCP characteristics.

**Table 1:** Sociodemographic and clinical characteristics of people living with brain tumour and healthcare professionals (HCPs)

People living with brain tumour		HCPs	
	*N* (%)		*N* (%)
**Median age**	51 years (IQR = 16.0)^a^	**Median age:**	43.5 years (IQR = 10.5)^b^
**Sex**	**24 (100)**	**Sex**	**11 (100)**
Male	10 (42)	Male	1 (9)
Female	13 (54)	Female	9 (82)
Not specified	1 (4)	Not specified	1 (9)
**Tumour type**^**c**^	**24 (100)**	**Primary clinical specialty**	**11 (100)**
Glioma	14 (58)	Oncology^d^	4 (36)
*Glioblastoma Multiforme* (*GBM*)	*4 (16)*	Nursing	3 (27)
Meningioma	4 (16)	Occupational therapy	2 (18)
Other^e^	5 (21)	Palliative care	1 (9)
Not specified	2 (8)	Not specified	1 (9)
**Tumour grade at diagnosis**	**24 (100)**	**Healthcare setting **^**e**^	**11 (100)**
Grade I	3 (12)	Hospital (inpatient)	2 (18)
Grade II–III	10 (42)	Hospital (outpatient)	8 (73)
Grade IV	4 (17)	Other^f^	2 (18)
Not specified	7 (29)	Not specified	1 (9)
**Years since diagnosis**	**24 (100)**	**Years of experience working with BT patients**	**11 (100)**
≤ 2	5 (21)	1 to 5	3 (27)
3 to 4	5 (21)	6 to 10	3 (27)
5 to 7	5 (21)	> 10	4 (36)
≥ 8	7 (29)	Not specified	1 (9)
Not specified	2 (8)		
**Treatment received**^**g**^	**24 (100)**	**Approximate number of BT patients seen each year**	**11 (100)**
Surgery	15 (63)	< 50	5 (45)
Chemotherapy	13 (54)	50 to 100	2 (18)
Radiotherapy	13 (54)	> 100	3 (27)
Not specified	3 (13)	Not specified	1 (9)
		**Most common tumour type seen**	**11 (100)**
		Glioblastoma multiforme (GBM)	10 (91)
		Not specified	1 (9)
		**Typical age profile of BT patients seen**	**11 (100)**
		50 to 65	8 (73)
		> 65	2 (18)
		Not specified	1 (9)

^a^One participant did not provide this information

^b^One participant had received two separate diagnoses 6 years apart

^c^Includes medical oncology, neuro-oncology and radiation oncology

^d^Includes oligoastrocytoma, atypical colloid cyst, mixed germ cell and ganglioneuroblastoma

^e^Multiple answers possible

^f^Includes community organisations and in-home rehabilitation

^g^Multiple answers possible

### Qualitative findings

Qualitative analysis identified one overarching theme and four themes, with seven subthemes pertaining to each theme. Themes/subthemes are described in-text and accompanied by illustrative quotes with unique participant identifiers (coded as P = person with BT, C = Caregiver, H = HCP). Results were corroborated by three member checking interviews conducted with people with BT (P4, P15, P24); duration of these interviews ranged from 37 to 67 min. See Table 2 for additional supporting quotes for each theme/subtheme.

**Table 2:** Illustrative quotes for themes and subthemes

Subtheme	Attribute		Quote	
Theme 1: Feedback on content				
*1.1. Relevance of content*	Positive aspects		‘I like that it’s more holistic that it’s including thinking about sleep, food, how to talk about it. I never thought about that’ [P12] ‘I actually liked how it sort of gave the support about how you might talk about it with other people, including that you don’t have to talk about it with other people if you don’t want to’. [H1]	
	Missing aspects		‘We think that routine would be a really good topic to discuss. Because I don’t think people in their forties and fifties would ever imagine you actually have to treat your partner like a baby’. [C2] ‘One of the things that we noticed was that there was no discussions around intimacy. And, you know, obviously, through a cancer journey, your intimacy changes’. [C1]	
	Sections needing more emphasis		‘The third session is work, home, and lifestyle assessment. That’s a lot. That’s a lot to process in one session’. [P29]	
	Irrelevant aspects		‘Anaemia is not something that needs to be on the list in this patient population…you need to have the cognitive things and the physical deficit things’. [H13] ‘It just depends on what age group you’re looking at too. Because work is not an issue for us, but it would be to somebody else’. [C4] *(i.e., need for flexible content)*	
*1.2. Appropriateness of goal setting*	For goal setting		‘I feel like goal setting or thinking about how to manage your symptoms is really important because it gives people more of a sense of something they can control, they can initiate, they can complete’. [P31]	
	Against goal setting		‘Setting goals and all that…the thought of it makes me even more tired’ [P21]	
	Not universally suitable		‘It depends what sort of diagnosis it is. If it’s a GBM and it’s stage four and all that, they may just not be up to sitting down and thinking about goals’. [H16]	
Theme 2: Feedback on format				
*2.1. User interaction and interface*	Positive aspects		‘I thought it was very user-friendly…Easy to navigate. There were no issues—it was seamless’ [H5]	
	Negative aspects		‘It takes you through too many processes. I think if we can try to cut down on those steps, that will be good’. [H11] ‘For a modern website, it is terrible… I think that the main problem with this website is it’s so dated. Because if it looks old, it feels like you’re getting into an old car. Do you want to get into an old car?’ [P1]	
	Use of language		‘I think it’s written in a way which is very accessible, so very simple language. Quite like it that it’s sort of bite-sized chunks’ [H3] ‘It was at a high health literacy level. I think for someone that has low literacy, it would be very difficult for them to engage with it’. [H11] ‘Some of the language is too removed from someone’s personal human experience. Like, so talking about SMART goals kind of makes you glaze over a little bit’. [C1]	
*2.2. Mode of delivery*	Variety in preferred mode		‘I would prefer online just because it’s easier for me to access it whenever I can rather than trying to fit in another appointment somewhere’ [P7] ‘I will always, every single time, choose hard copy information over anything on the computer’. [C2] ‘When you’re having so much fatigue and brain fog in life, you’re so isolated. And so… it’d be great if there was a support group connected to this in some way’. [P5] ‘When you’re recovering, you know, your mind isn’t that great for memory so… if it was part of an app, you could then put into it reminders and all that type of stuff’ [P33]	
	Desire for audio-visual components		‘I have moments where I’m so tired I can’t read [and] where a video would be better’. [P12]	
	Desire for multiple options		‘I think you have to have multiple options’. [H13] ‘Everyone is different. We have all different kind of days and I think it’s good if we have different options’. [P12]	
Theme 3: Feedback on use				
*3.1. Preferences for assistance*	Dependent on the individual		‘Some patients would be able to do it quite easily by themselves. Some patients would need a carer or somebody to do it with them. So I think again it depends on who they are’. [H8]	
	Caregiver involvement		‘If you put the patient carer dyad together… you give benefit to the patient, you get better benefit to the carer and you get that sense of solidarity that they’re doing it together’. [H7] ‘I think anyone with brain cancer whose partner is sticking by them, can only benefit from having the partner fully, fully involved in the journey’. [P2]	
	HCP involvement		‘There is something really lovely about having that option of… a health provider working with people, up to five or six people, and helping each person work out a goal’. [P5]	
	Support network involvement		‘If we just do it ourselves, we can kid ourselves that everything’s okay but if I make other people aware “No, that’s not right you are not coping.”’ [P10]	
*3.2. Optimal timing*	Introduce early		‘For somebody right back in the beginning to be given the information that you hopefully will be able to deliver—I think it would have made a massive difference’. [C2]	
	Introduce after first-line treatment		‘First three months you’re kind of like just trying to work out literally whether you can walk or not… You don’t know you’re a mess but you’re a mess, so you’re not capable of absorbing anything’. [P8]	
	Offer at regular intervals		‘Leave it in the hands of the patient, but keep asking. You know, so they might not be ready today, but they might be ready six months from now…So when you say how quickly, I would say immediately, but also repetitively’. [P29]	
*3.3. RESTORE as a resource for caregivers, family and friends*	As an educational resource		‘It’s really important for families to understand about fatigue…often they will be the ones who need to have the understanding so they can help support the person managing it better’. [H12]	
	To support caregiver fatigue		‘This is really the first I’ve really, even for myself, thought about my own sleep patterns and what I get up to as well’. [C2] ‘If you target fatigue for both patients and carers, I think you have a potential synergistic effect’. [H7]	
	As a conversation starter		‘It’s just a hard topic at times when you’re going through so much. This kind of just breaks the ice a bit, enables that discussion’. [P33]	
Theme 4: Barriers to engagement				
		Tumour-related deficits		‘For somebody, for example, who’s got pretty progressive disease in the frontal area, who’s got no insight and is pretty reliant on their family… I would say, look, you’re just sort of flogging a dead horse there’. [H5] ‘Understanding what the deficits are I think is going to be the crucial element in terms of whether or not they will engage with the project’. [H7]
		Caregiver burden		‘There absolutely comes a time where the carer just does not have the time’. [C2]
		Digital access and literacy		‘We do have patients who are very low tech and this will obviously not work for them at all because they don’t even have an email account’. [H12] ‘I live quite remotely… if I’m at home, which is out of town by about four kilometres, I couldn’t do a video like this’. [P46]
		Personality		‘I thought that [goal setting] was interesting, but it’s not something that I do. Because I’m too lazy’. [P16]
		Awareness of the program		‘The barrier would be making them aware that it existed’. [H4]
		Diversity of people with BT		‘If you’ve got a high-grade brain tumour and your survival’s 16 months, you’re probably not going to utilise this as much I think’ [H4]
				‘It needs to be individually tailored’. [P17] ‘I think the problem here is that there’s probably no one answer that suits absolutely everybody’ [H1]

### Overarching theme—need for flexible and responsive tailoring

All HCPs said they would recommend a modified BT-specific version of RESTORE, and most patients and caregivers said they would be willing to use it to address fatigue. However, all feedback was underpinned by the belief that generic modifications would not be sufficient to address the diversity among people with BT. Rather, the program would require flexible and responsive tailoring to individual needs and characteristics.

### Theme 1—feedback on content

#### Relevance of content

Overall, participants found RESTORE content relevant, comprehensive and helpful. Participants particularly liked the fatigue diary and section on ‘Talking to Others’. However, they stated several aspects were irrelevant, missing or needed greater emphasis. For example, participants indicated the ‘Work, Home & Lifestyle’ session was ‘a lot to process in one session’ [P29] and suggested breaking it into three separate, optional sections, especially given some people with BT may no longer work. In the session covering causes of fatigue, some HCPs noted ‘anaemia is not something that needs to be on the list for this population’ [H13]. Instead, they suggested covering causes of fatigue that are more relevant to people with BT such as cognitive difficulties, medications, or specific BT location. Other aspects considered missing included information on fatigue-related sexual changes and intimacy, content related to different life stages (e.g. retirement) and emphasis on the importance of routine in managing fatigue.

Several participants acknowledged aspects not relevant to them may be to others (e.g. ‘work is not an issue for us, but it would be to somebody else’ [C4]), emphasising the need for flexible and layered delivery of content according to individuals’ needs.

#### Appropriateness of goal setting

Most participants found goal setting a helpful strategy for managing fatigue. For example, some people with BT believed it could help them regain a sense of control and enhance motivation to take steps to manage fatigue. However, several participants noted goal setting could be challenging for those with severe cognitive difficulties or poor health/prognosis. Some participants supported this stating they thought goal setting could be an ‘overwhelming’ [H7] and ‘really tiring’ [P21] experience that could induce ‘failure guilt’ [H7]. To overcome this, participants suggested involvement of a support person and incorporating greater flexibility with the amount of goal setting required.

### Theme 2—feedback on format

#### User interaction and interface

Generally, participants thought RESTORE was user-friendly and ‘easy to navigate’ [H5]. To improve the user interface, participants suggested updating the website’s design and reducing the amount of text by incorporating more images and diagrams relevant to people with BT. Participants liked the modular structure of RESTORE, although some noted the amount of ‘clicking’ required could be cumbersome for people with BT. To overcome this, some participants suggested allowing users to freely navigate within and between sessions (e.g. by including a contents bar).

There was mixed feedback on the language used. Some believed it was ‘clear and very easy to understand’ [P34], whereas others said it was ‘at a high health literacy level’ [H11]. Some participants felt the language was ‘patronising’ [P13], ‘removed’ [C1] or ‘too academic’ [H17]. These views were especially evident regarding the language around SMART goals. Similar sentiments were raised regarding the fatigue quiz in session 1, with some participants commenting it made them feel ‘really irritated’ [H7], ‘told off’ [P13], and ‘tested’ [H17].

#### Mode of delivery

Most participants preferred RESTORE in its current, online format. Others noted preferences for hard copy materials, a mobile device-enabled app, face-to-face sessions, or podcasts. Regardless of personal preference, most participants acknowledged the need for multiple delivery options to cater for the broad spectrum of needs because ‘everyone is different’ [P12]. Many people with BT found reading text-based materials fatiguing and desired more audio-visual content.

### Theme 3—feedback on use

#### Preferences for assistance

Most participants stated assistance would be required for people with BT to use RESTORE. Some believed caregivers would be best placed to provide this support, with many noting their involvement could have mutual benefits and bolster patient-caregiver ‘sense of solidarity’ [H7]. Finally, some participants suggested a ‘support network’ of people could potentially encourage use of RESTORE and provide accountability and encouragement (e.g. trained volunteers, local community, or a RESTORE-specific support group).

#### Optimal timing

Most participants felt RESTORE should be introduced early (e.g. ‘day one’ post diagnosis [P10]), so both people with BT and caregivers knew what to expect about CRF. However, others were concerned introducing the program too early could be overwhelming. To address this concern, some HCPs suggested introducing RESTORE after first-line of treatment (e.g. after radiation therapy). Others suggested there was no optimal timing and recommended reminding patients and caregivers about the availability of RESTORE at regular intervals throughout the disease and treatment trajectory.

#### RESTORE as a resource for caregivers, family and friends

Most participants believed RESTORE could be a useful resource for the caregivers, family and friends of people with BT. Some thought it could help ‘break the ice’ [P33] by ‘opening up a conversation’ [C2] about the extent and impact of CRF. Others noted RESTORE could also help address caregiver fatigue, with some caregivers reporting it made them reflect on their own fatigue and management strategies. Some HCPs also noted RESTORE could provide mutual benefits to the person with BT and caregiver when completed together. However, this would require modifications to allow caregivers’ to easily review the content without having to actively participate in the intervention (e.g. inclusion of a ‘skip’ or ‘I’m not a patient’ button).

### Theme 4—barriers to engagement

Participants identified several barriers which may limit an individual’s ability or willingness to engage with RESTORE. Core barriers included (1) tumour-related cognitive deficits, (2) caregiver burden, (3) digital access and literacy, (4) personality traits, (5) awareness and (6) diversity of people with BT.

Several participants, mostly HCPs, noted people with BT with tumour-related cognitive deficits may struggle to engage with RESTORE and would likely need assistance. In turn, caregiver burden was also identified as a potential barrier for patients who need support.

As RESTORE is currently only offered online, several participants noted it may not be accessible to demographic groups who are ‘not tech savvy’ [H11]. Participants commonly raised computer/internet access as a restriction on individuals’ ability to use RESTORE, especially for those living in regional or remote areas.

Some participants felt personality traits may reduce a person’s willingness to engage in the program particularly due to the emphasis on goal setting, with some people with BT claiming they were not goal oriented.

Even when participants were willing to engage with RESTORE, some questioned ‘How would people find out about it?’ [P38]. Likewise, some HCPs were concerned RESTORE would be lost among other information resources and ‘pushed to the back of [patient’s] list of reading’ [H3].

One of the greatest barriers to implementing an effective BT-specific version of RESTORE is that ‘it’s really difficult to be useful generically’ [P13]. Since ‘there isn’t just one type of patient’ [P24], participants reported RESTORE needed to be flexible and provide layered information to address the diversity of symptoms and needs in this population.

As per interpretive description methodology [24], identified themes and subthemes were interpreted to devise a series of recommended changes. See Table 3 for the full list of suggested modifications to create a BT-specific version of RESTORE. In brief, these included provision of narratives from people with BT; inclusion of accessibility features (e.g. video and audio content); discussion of causes of fatigue that are more specific to BT; changing language around SMART goals; reducing text and length of sessions; and encouraging involvement of a support person.

**Table 3:** Proposed modifications to RESTORE for people with BT based on participant feedback

Relevant section of RESTORE	Issue	Suggested Modification
Session 1: *Introduction*	Causes such as anaemia and pain are less relevant to BT compared to other cancers. Instead, cognitive difficulties and BT location may be more relevant to this population	Make the causes of fatigue page specific to BT patients
	Fatigue-related sexual changes were a significant issue missing from RESTORE	Include content on intimacy and sexual changes
	Impacts of drugs on fatigue (both good and bad) could be included in the fatigue education section	Include information on the effect of pharmaceuticals on fatigue
	Mixed responses to being quizzed on own condition, especially when a large red cross appeared if incorrect answers supplied. Suggested adjusting to ‘Guess how much’ or ‘What do you think?’ rather than a test, especially as relevant statistics has not been covered previously	Modify/remove the quiz from Fatigue Education
	Some would or could not print the diary, reducing its use and therefore its value	Create an online version of the fatigue diary
Session 2: *Goal setting*	Goal setting was expected too often and could be overwhelming	Allow for flexibility in the amount of goal setting required
	To help goal setting, provide example goals relating to common brain tumour concerns	Provide BT-specific examples of goal setting
	RESTORE catered to recently diagnosed patients who had not attempted goal setting before. There is a need for information on how to set goals as a long term-survivor (e.g. after previous failed attempts)	Adjust goal-setting information to cater to longer-term survivors
	Goal setting may be difficult for some people with BT due to cognitive deficits	Encourage the involvement of a support person when setting goals
	SMART goals were too simplistic, patronising, and/or academic. Make the language more personal and conversational to help people with BT set goals and minimise guilt or discouragement if goals were not achieved	Change language around SMART goals (e.g. ‘What’s important to you?’)
Session 3: *Work, home and lifestyle*	Importance of routine in managing fatigue	Include information on the effectiveness of routine
	Work, home, and lifestyle were all important topics that deserved their own detailed sections. Also indicated the need for greater chunking of information	Break down session 3 into separate, optional sections
Session 5: *Talking to others*	RESTORE assumes positive outcomes when talking to an employer which is not a universal experience	Adjust language in ‘Talking to Others’ to reflect all possible outcomes
	Communicating needs to loved ones was reported to be challenging. Desire for examples of how to do this so their loved ones could offer better support	Provide example scripts to guide people with BT through difficult conversations with others
General	Information is UK-based and would need to be adjusted to the Australian context	Change information from UK to Australian context
	Survivor vignettes were valued but would be more beneficial to have vignettes specific to common BT difficulties	Include BT-specific vignettes
	Links to support groups, information resources and allied HCPs to address specific BT needs were requested	Provide links to other resources, support groups and allied HCPs
	Content for older adults was missing or sometimes irrelevant (e.g. work)	Include information for older demographics
	Getting to end of RESTORE felt abrupt and unclear	Make the completion of RESTORE clear
	Landing page was outdated and difficult to navigate (e.g. lots of clicking)	Improve navigation from home page
	Website navigation could be improved (e.g. the amount of clicking involved is cumbersome for those already fatigued). Reduce this burden by providing flexible navigation throughout the intervention	Allow users to freely navigate within sessions (e.g. provide a contents bar along the side)
	Length of each session could be overwhelming and potentially lead to drop-out. Shorter sessions were preferred	Reduce the length of each session
	Progress is presented (e.g. 0 of 22 pages) in a way that makes the intervention seem content-heavy and potentially overwhelming for patients	Modify the way current progress is presented (e.g. percentage completed)
	Website was dull and unengaging and might lead to fewer people using the resource. Current design lacks diversity	Update visuals (e.g. more graphics, change colour palette, include images of people with BT)
	Preference for video and/or audio components within RESTORE, especially given the common cognitive deficits experienced by many people with BT	Include video and/or audio components (e.g. video rather than written patient vignettes)
	RESTORE was too text heavy and didn’t have enough graphics	Reduce the amount of text (e.g. add images, segment information)
	People with lower literacy levels, cognitive difficulties or extreme fatigue may struggle with the intervention due to the language complexity. Language perceived by some as ‘scientific’ and ‘too removed from someone’s personal human experience’	Personalise and simplify language
	Unclear what ‘recent’ fatigue referred to in the self-assessment and preference for a concrete timeframe. Some found it difficult to self-assess fatigue if they you didn’t know what to compare it to	Clarify definitions (e.g. what is ‘recent’)
	Challenge with fatigue assessments as the ‘negative’ and ‘positive’ end for each were opposite-concern that this could lead to incorrect responses	Make direction of fatigue measures consistent
	At the moment, participants must complete the fatigue ratings in each session. Consider including a ‘skip’ or ‘I’m not a patient’ option, so HCPs and caregivers can review the content completed by the patient without having to actively participate in the intervention	Make the interactive components of RESTORE optional so HCPs and caregivers can easily review the content
	Some of the content did not transfer between a mobile device vs laptop	Ensure that RESTORE is properly formatted across all possible electronic devices
	Regardless of personal preference, there is a need for RESTORE to be offered via different modes of delivery to cater for the broad range of people with BT and caregivers	Offer multiple delivery options

*Ps* participants, *BT* brain tumour, *HCP* healthcare professional

## Discussion

This study explored the appropriateness of a web-based intervention (RESTORE) to support self-management of fatigue for people with BT among a diverse group of stakeholders. Overall, participants expressed the desire for a BT-specific version of RESTORE and identified several positive features of the intervention, such as provision of support for talking to others and tools for monitoring fatigue. However, several shortcomings of the content and format were identified, as well as potential barriers to engagement, reducing its usefulness to the BT population. To optimise effectiveness for people with BT, these limitations and barriers need to be addressed in the development and implementation of a BT-specific version of RESTORE.

In line with recent calls for behaviour change interventions to be personalised to optimise effectiveness and translation into routine cancer care [32], a common thread emerging throughout was the overarching need for flexibility in intervention content and delivery responsive to individual patient needs. Given the diversity of people with BT, in terms of subtypes and prognosis, age, stage of survivorship, and working status, there is a need for RESTORE content to be layered so the end-user is not required to navigate all content, but can opt to engage with topics directly relevant to them, minimising cognitive burden and user fatigue. Participants also noted a need for content to be more relevant to their lived experience by providing information specific to brain tumour (sub)types and their geographic region. As the RESTORE intervention was originally developed for the UK context [20], the images, videos, and links to other resources need to be tailored to local contexts for use in other countries internationally.

Similar to findings from a previous review of self-management support for cancer-related fatigue [33], participants indicated diverse preferences for mode and timing of intervention delivery as well as completion assistance. This suggests where possible self-management interventions should be offered in multiple formats (e.g. hard copy, online, face-to-face) at regular intervals throughout the disease and treatment trajectory, with the option to complete alone or with assistance, to cater for individual patient preference and needs. To facilitate this, future work should identify the minimum number of alternative modes of delivery required to maximise engagement.

Our findings indicate mixed views on the usefulness of SMART goals to promote self-management of fatigue in people with BT. Although some participants noted potential benefits, others felt this approach may be overwhelming, engendering feelings of failure if goals are not met, also noting concerns with the complexity of language-used around SMART goals. Supporting this, previous research examining use of SMART goals for physical activity promotion found they can lead to negative outcomes for insufficiently active individuals, including less physical activity, enjoyment, pleasure, motivation, and significantly greater pressure/tension [34, 35]. As a result, their use has been brought into question [36]. Our results similarly suggest use of SMART goals to promote self-management of fatigue may not be universally appropriate for this population, warranting exploration of alternative techniques to support behaviour change in people with BT such as action planning and motivational interviewing [37].

Results further indicated RESTORE could be useful more broadly as a resource for carers, family members and friends of people with BT to educate them on what CRF is and how it impacts on the person with a BT. Given previous research consistently found CRF is under-assessed and under-treated in clinical settings [3, 10, 11], this raises the question as to whether RESTORE could be a useful educational resource for HCPs to reinforce awareness around the importance of routinely assessing and discussing CRF management with people with BT.

Several barriers to engaging with RESTORE were identified and need to be addressed to maximise uptake among people with BT. These barriers include specific patient characteristics (tumour-related cognitive deficits, digital literacy, personality traits), issues with digital access, lack of awareness of the intervention, and burden on caregivers when people with BT need assistance with RESTORE. Of note, hardcopy information booklets on CRF are available at Cancer Information Centres; these may be useful for those who lack online access or computer literacy. Nevertheless, previous research identified a lack of CRF information resources specific to people with BT [3], emphasising the need for tailored CRF resources for this population regardless of mode of delivery. Although certain patient characteristics may be unavoidable and preclude effective engagement with a BT-specific version of RESTORE, other barriers can be addressed by offering RESTORE in an additional ‘offline’ format (e.g. hardcopy), reducing the literacy level, providing RESTORE in a guided rather than self-directed format and implementing regular screening of fatigue in clinical practice settings with automated referral to RESTORE.

Overall, our findings indicate even with BT-specific adaptations, RESTORE is unlikely to be suitable for all people with BT. Specifically, RESTORE is unlikely to be appropriate for those with severe cognitive difficulties, rapidly deteriorating, or at end of life. Our results emphasise, given the diversity of people with BT, it is challenging to create a ‘one size fits all’ self-management intervention to address fatigue. Nevertheless, our findings suggest a BT-specific version of RESTORE is likely to be appropriate for at least a proportion of people with BT, especially those without significant cognitive and physical deficits who are motivated to self-manage their fatigue. Moreover, given people with moderate to severe CRF with other cancer types report significant disability [38] and may have difficulty completing online programs independently, many of the recommended modifications to RESTORE for the BT population may be appropriate to optimise RESTORE for other cancer survivors with clinically significant CRF.

Despite garnering perspectives from diverse stakeholders with lived or professional experience with BT, this study has some limitations. All participants were recruited from Australia only, limiting generalisability of findings to people with BT in other countries and cultures. In addition, there was relatively limited participation of HCPs and BT caregivers. This is a problem inherent to conducting research in these groups due to time constraints and demands on both HCPs and BT caregivers. Sampling bias may also be at play given people with BT who volunteered to participate in this study were those with sufficient cognitive and technical capacity to participate in a one-hour interview via videoconference and review RESTORE online. Hence, perspectives and feedback are likely to differ from people with BT with more significant physical and cognitive deficits and limited technical access/abilities. As further development of a BT-specific version of RESTORE occurs, efforts should be undertaken to obtain feedback from these individuals through use of alternative consent processes [39] and enabling them to trial the adapted materials for short periods of time. Finally, all participants were English-speaking with limited participation of individuals from culturally and linguistically diverse backgrounds. Future research is needed to explore whether further tailoring of a BT-specific version of RESTORE is required for these groups.

## Conclusion

People with BT, caregivers, and HCPs expressed a desire for a BT-specific version of RESTORE to support self-management of fatigue. Adaption of the existing version of RESTORE is acceptable, provided the intervention is modified to provide greater flexibility and tailoring to BT-specific needs. By engaging in a co-design process with key stakeholders to identify appropriate adaptions to RESTORE, these results will maximise the utility of the intervention for this population to ensure effective engagement. Although RESTORE may only be appropriate for a subgroup of people with BT, given its acceptability and the paucity of interventions available for this population, a BT-specific version of RESTORE warrants further investigation and evaluation.

## Supplementary Information

Below is the link to the electronic supplementary material.

Supplementary file1 (DOCX 45 KB)

## Data Availability

The datasets generated during and analysed during the current study are available from the corresponding author on reasonable request.

## Footnotes

1 Part 2 of the interview guide was used to address the objectives of this study. Findings relating to Part 1 have been previously published [3].
